# Exciton Dynamics in MoS_2_-Pentacene and WSe_2_-Pentacene Heterojunctions

**DOI:** 10.1021/acsnano.2c06144

**Published:** 2022-09-30

**Authors:** Pavel A. Markeev, Emad Najafidehaghani, Gergely F. Samu, Krisztina Sarosi, Sirri Batuhan Kalkan, Ziyang Gan, Antony George, Veronika Reisner, Karoly Mogyorosi, Viktor Chikan, Bert Nickel, Andrey Turchanin, Michel P. de Jong

**Affiliations:** † MESA+ Institute for Nanotechnology, University of Twente, 7500 AEEnschede, The Netherlands; ‡ Institute of Physical Chemistry, Abbe Center of Photonics, Friedrich Schiller University, 07743Jena, Germany; § ELI-ALPS, ELI-HU Non-Profit Ltd., Wolfgang Sandner 3, SzegedH-6728, Hungary; ∥ Faculty of Physics and CeNS, Ludwig-Maximilians-Universität, Geschwister-Scholl-Platz 1, 80539Munich, Germany; ⊥ Department of Chemistry, Kansas State University, 213 CBC Building, Manhattan, Kansas66506-0401, United States

**Keywords:** transition metal dichalcogenides, organic semiconductors, pentacene, exciton dynamics, transient reflection spectroscopy, MoS_2_, WSe_2_

## Abstract

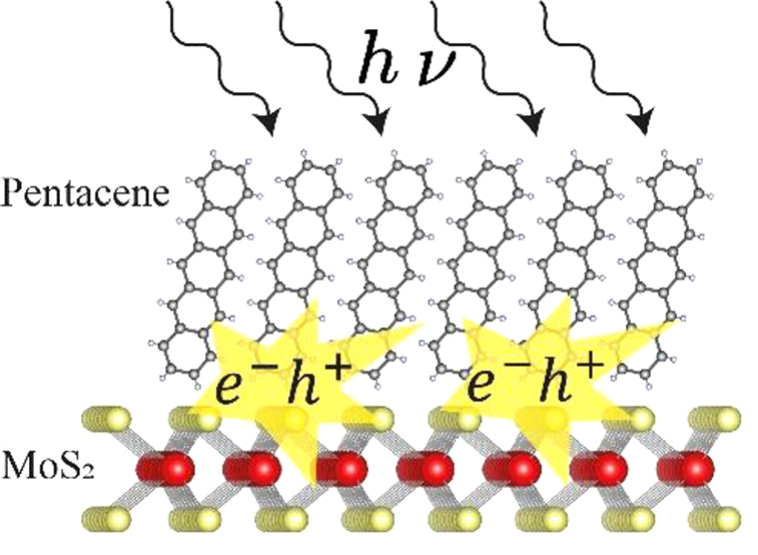

We measured the exciton dynamics in van der Waals heterojunctions of transition metal dichalcogenides (TMDCs) and organic semiconductors (OSs). TMDCs and OSs are semiconducting materials with rich and highly diverse optical and electronic properties. Their heterostructures, exhibiting van der Waals bonding at their interfaces, can be utilized in the field of optoelectronics and photovoltaics. Two types of heterojunctions, MoS_2_-pentacene and WSe_2_-pentacene, were prepared by layer transfer of 20 nm pentacene thin films as well as MoS_2_ and WSe_2_ monolayer crystals onto Au surfaces. The samples were studied by means of transient absorption spectroscopy in the reflectance mode. We found that A-exciton decay by hole transfer from MoS_2_ to pentacene occurs with a characteristic time of 21 ± 3 ps. This is slow compared to previously reported hole transfer times of 6.7 ps in MoS_2_-pentacene junctions formed by vapor deposition of pentacene molecules onto MoS_2_ on SiO_2_. The B-exciton decay in WSe_2_ shows faster hole transfer rates for WSe_2_-pentacene heterojunctions, with a characteristic time of 7 ± 1 ps. The A-exciton in WSe_2_ also decays faster due to the presence of a pentacene overlayer; however, fitting the decay traces did not allow for the unambiguous assignment of the associated decay time. Our work provides important insights into excitonic dynamics in the growing field of TMDC-OS heterojunctions.

## 1 Introduction

The study of van der Waals heterojunctions based on transition metal dichalcogenides (TMDCs) and organic semiconductor (OS) thin films has been ongoing for several years; however, there are still many fascinating avenues for researchers to explore.^1^ The TMDCs themselves exhibit fascinating physical phenomena such as exceptionally large exciton binding energies,^2,3^ spin-valley locking,^4^ and the exciton Hall effect.^5^ TMDCs and organic semiconductors are highly dissimilar nonconventional semiconductors that share the characteristic of interlayer bonding via van der Waals interactions. Forming heterojunctions of these materials offers interesting opportunities for combining diverse (opto-)electronic properties. An important step for the technological development of such structures has been taken recently, in the demonstration by some of us of devices fabricated by layer transfer of TMCDs as well as organic semiconductor thin films.^6^ A strong advantage of this approach is that the organic semiconductors can be grown on a suitable substrate for obtaining high quality crystalline layers, avoiding molecular disorder that occurs upon direct deposition on TMCD substrates.

The wide variety of devices that have been constructed in the field so far include light emitting diodes,^7^ tunneling transistors,^8^ and photovoltaic cells.^9,10^ Arguably, one of the most promising applications out of these possibilities is photovoltaics, and the present work is carried out in its context, focusing on exciton dynamics at TMDC/OS interfaces. Some of the aforementioned heterojunctions have been studied previously in various combinations and conditions by similar ultrafast pump probe spectroscopic techniques.^10−16^ Most of these previous studies focused on 2D TMDCs grown on quartz and SiO_2_/Si wafers, which were then covered by an OS layer directly deposited onto the TMDC monolayers. TMDCs such as MoS_2_ and WSe_2_ monolayers have attracted a lot of attention as promising 2D semiconductors and have been studied thoroughly.^17−20^ Here, we examine high quality monolayers of MoS_2_ and WSe_2_ prepared by a dedicated chemical vapor deposition (CVD) technique and transferred to a UV ozone treated Au substrate.^21−23^ Homogeneous pentacene films, 20 nm in thickness, were also transferred to the samples after uniform controlled growth on poly(acrylic acid) (PAA) substrate films.^6^

Transient absorption/reflection spectroscopy (TAS/TRS) is a well-known technique to study ultrafast processes of exciton formation and dissociation in various materials.^12,24,25^ Even though the performance of photovoltaic devices is based on many different aspects, one of these is the temporal difference between exciton decay and charge separation on the junction interface. Recently, relatively long lasting (5.1 ns) charge-separated states at MoS_2_-pentacene interfaces have been reported.^15^ In this work, pentacene was directly grown on the surface of MoS_2_, while MoS_2_ was deposited on the substrate by a CVD method similar to ours. Here, we focus on the ultrafast exciton dynamics in van der Waals heterojunctions of WSe_2_ and MoS_2_ monolayer crystals covered by a 20 nm transferred thin film of pentacene. This allows us to investigate to which extent molecular (dis)order at the MoS_2_/pentacene interface affects exciton dissociation rates. Charge transfer dynamics at WSe_2_/pentacene interfaces have not been studied previously.

## 2 Results and Discussion

Here, the obtained TRS results are presented in the following manner. First, we discuss the pentacene reflection spectra. Next, TRS results of MoS_2_ and MoS_2_-pentacene heterojunctions are presented, with analysis and conclusions. Lastly, the ultrafast spectroscopic analysis of the results of WSe_2_ and WSe_2_-pentacene composites are presented and discussed.

**Figure 1:**
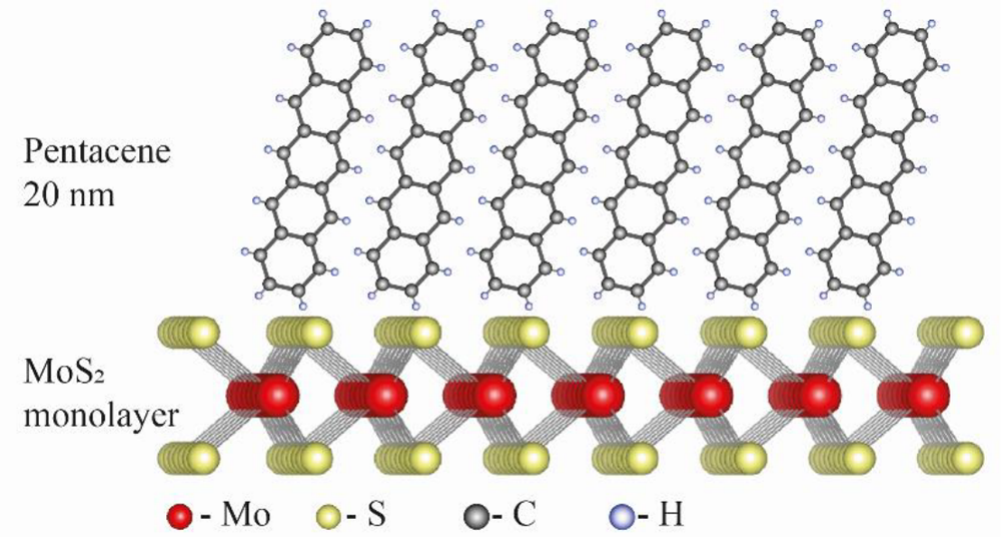
Structural arrangement of pentacene on MoS_2_.

Figure 1 shows the structural arrangement of pentacene molecules on MoS_2_. We expect highly oriented vertically packed pentacene molecules at the interface with the TMDC monolayer. The same arrangement is expected for the WSe_2_-pentacene sample. Photoemission electron microscopy (PEEM) images of similar samples, showing the crystalline nature of the pentacene layer, can be found in Figure S6 in the Supporting Information.

### 2.1 Pentacene

**Figure 2:**
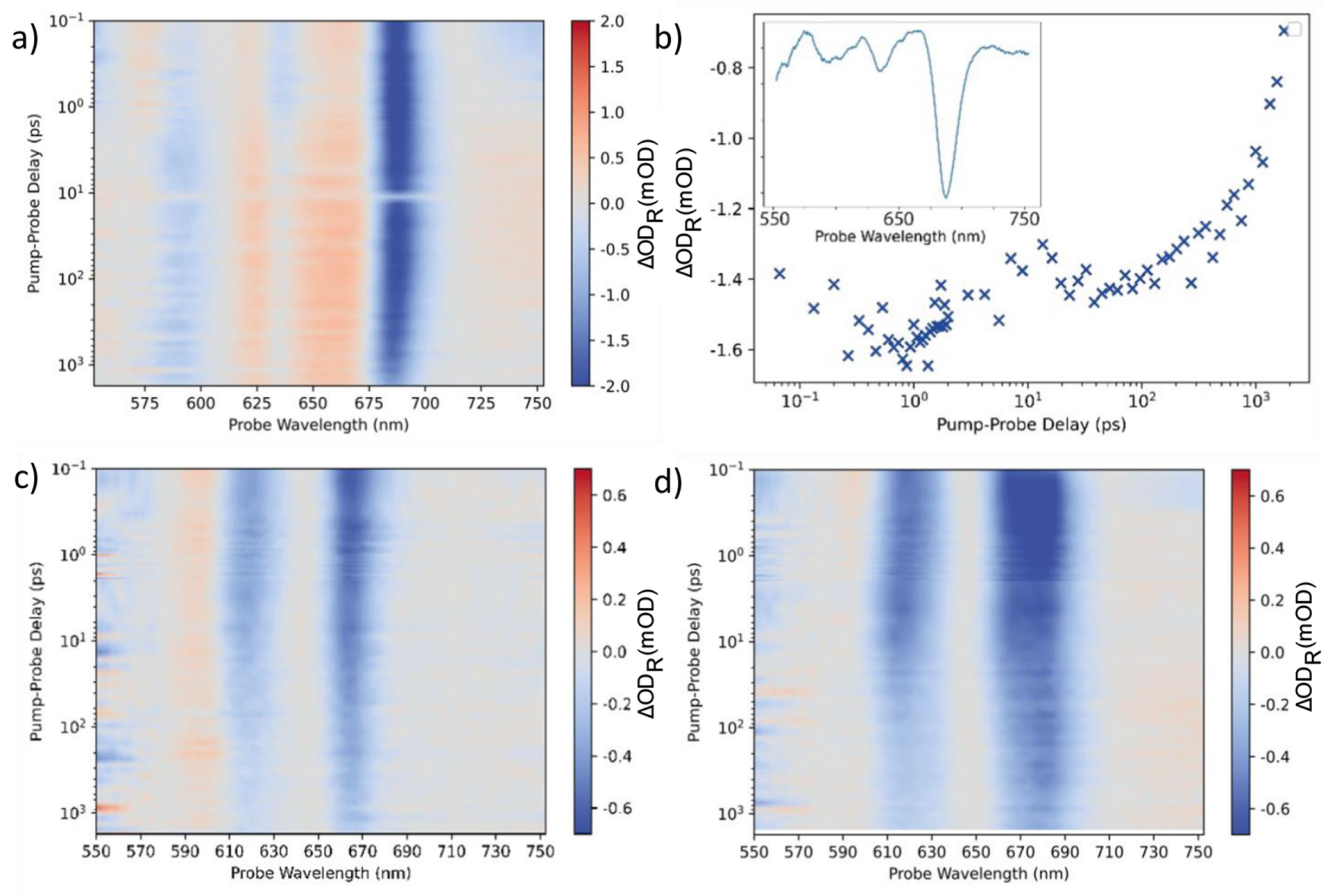
(a) Two-dimensional transient reflection spectra of a 20 nm pentacene film on a gold substrate. Here and further the background subtraction (described in Supporting Information 2) is applied to this type of result. (b) A kinetic trace of the main feature at 675–695 nm from the averaged spectra in (a); inset represents the spectrum at 0.53 ps delay time. (c, d) Two-dimensional transient reflection spectra of (c) MoS_2_ monolayer crystals transferred to a gold substrate and (d) similar MoS_2_ monolayer crystals on gold covered by a pentacene thin film.

Figure 2a shows two-dimensional (2D) absorption spectra (average of measurements taken at three different locations) on a 20 nm thin film of pentacene transferred to gold. The sample is photoexcited at 515 nm, and the transient reflectivity spectra are recorded. ΔOD_R_ is calculated as −log(*I*_R,exc_/*I*_R,noexc_) where *I*_R,exc_ and *I*_R,noexc_ are the reflected light intensity with and without excitation, respectively. The temporal resolution of the experiment is 80 fs. The spectra are dominated by a strong ground state bleach feature, attributed to the depopulation of the ground state by the pump pulse. This feature is centered at 688 nm and corresponds to the lowest energy singlet exciton in pentacene. Two weaker bleach signals are recognizable at 635 and 597 nm. The spectrum recorded at a pump–probe delay time of 0.53 ps can be found in the inset of Figure 2b. These results are in good agreement with previously published works on (transient) optical absorption spectroscopy of (poly)crystalline pentacene.^25−27^ The separation between the 688 nm (1.80 eV) and 635 nm (1.95 eV) peaks can be attributed to the Davydov splitting due to the presence of two inequivalent pentacene molecules in the unit cell.^27^

Figure 2b shows the kinetic trace extracted at the main bleach feature of the pentacene film (integrated signal intensity from 675 to 695 nm). The data show that the ground state bleach is long-lived and hardly evolves within 1 ns after excitation. Note that ultrafast (sub 150 fs) fission of singlet excitons into two triplet excitons, which is known to be an efficient process in pentacene,^25,26^ is not detectable on the comparatively long time scales probed in our measurements.

### 2.2 Molybdenum Disulfide (MoS_2_)

**Figure 3:**
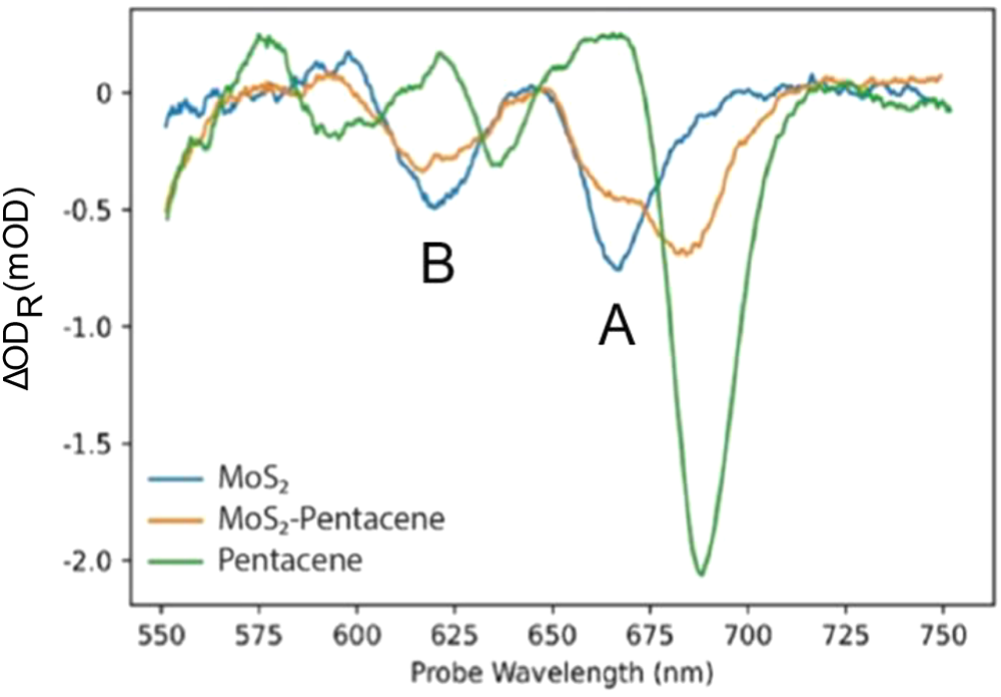
Transient reflection spectra of a MoS_2_ monolayer, a MoS_2_-pentacene junction, and a pentacene thin film (all on Au substrates) probed at 0.53 ps.

Figure 2c,d shows transient reflection spectra for uncovered MoS_2_ single crystals on Au (Figure 2c), as well as MoS_2_ monolayer crystals on Au that are covered by a 20 nm continuous film of pentacene (Figure 2d). There are two main ground state bleach peaks observed for the MoS_2_ single crystals, centered at 615 nm and at 662 nm. These values represent the B- and A-excitons of MoS_2_, respectively. The decay processes that these excitons undergo in similar samples (i.e., MoS_2_ monolayers on amorphous SiO_2_, quartz and sapphire) have been thoroughly studied previously.^20,28−30^ For pentacene-covered MoS_2_ (see Figure 2d and Figure 3), two partially overlapping ground state bleach features appear at about 670 nm, which are attributed to the MoS_2_ A-exciton and the closely located pentacene bleach peak at 688 nm.

Figure 3 shows the transient reflection spectra of three different samples: the MoS_2_ monolayer in blue, pentacene in green, and the MoS_2_ crystal covered with the pentacene thin film in orange. These spectra were captured 0.53 ps after photoexcitation. As was discussed earlier, pentacene has three main bleach peaks, and MoS_2_ has two, while the intense pentacene ground state bleach at 688 nm is located very close to the MoS_2_ A-exciton peak. In the MoS_2_-pentacene data, one can observe a composite feature that results from the superposition of the two individual peaks.

To study the mechanism(s) of exciton decay in the kinetic traces and to provide quantitative comparison of the various samples, we fitted the data with a triple exponential decay function as shown below:
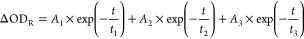
1

**Figure 4:**
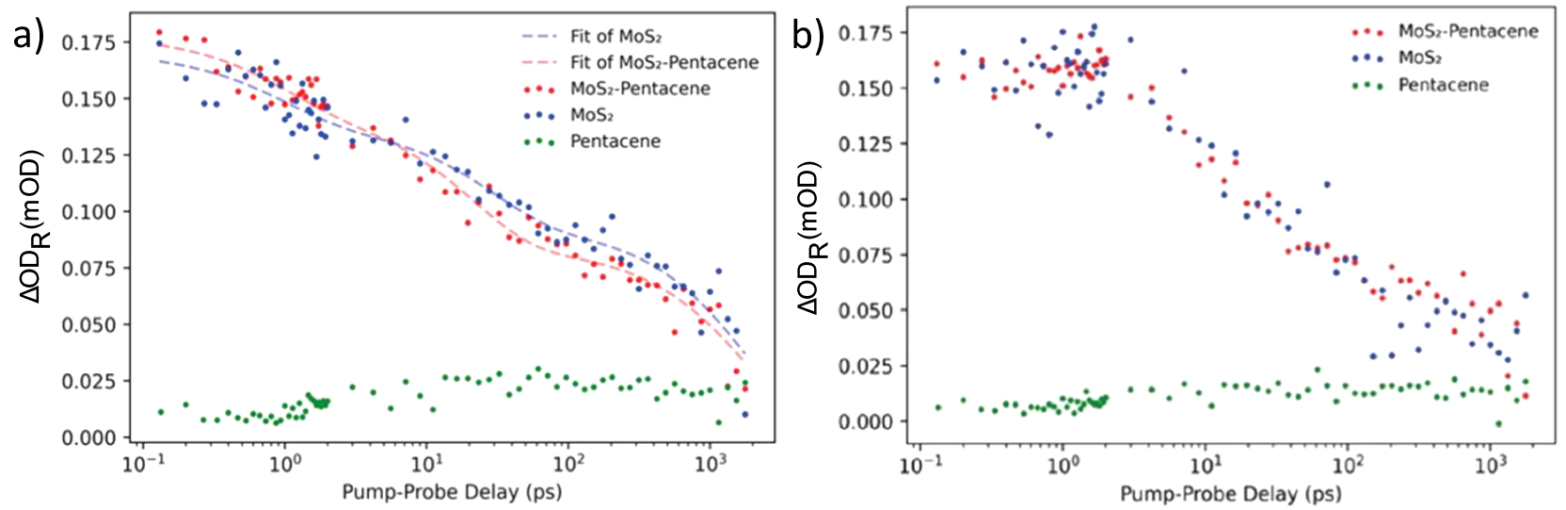
Normalized decay traces of MoS_2_ excitons in a MoS_2_ monolayer compared to those in a MoS_2_-pentacene junction, both on gold substrates. (a) A-exciton probed at 665 nm, fitted by a three-exponential decay function; (b) B-exciton probed at 615 nm.

The results of this fitting can be seen in Figure 4a, corresponding to the kinetic trace of the A-exciton of MoS_2_. The fast decay component of the A-exciton in uncovered MoS_2_ crystals was fitted to be 1.0 ± 0.5 ps. This component is usually attributed to fast trapping processes of excitons at intrinsic defect sites such as atomic S- and S_2_-vacancies,^15,29,31,32^ and/or to exciton cooling effects.^33−35^ The second component of 28.3 ± 9.4 ps represents the nonradiative decay of exciton–phonon pairs.^30,32^ The slow component of 1.9 ± 0.3 ns is usually associated with the lifetime of free charges^35^ and their radiative recombination.^36,37^ The fractional weight of each decay component is presented in Table 1 next to the values of the decay times.

**Table 1:** Fit Parameters for Decay Traces of A-Excitons in Composite MoS_2_ and MoS_2_-Pentacene Samples^a^

	*t*_1_ [ps] (A1)	*t*_2_ [ps] (A2)	*t*_3_ [ps] (A3)	*t*_4_ [ps] (A4)
	carrier trapping	h^+^ transfer	exciton–phonon scattering	radiative recombination and e^–^ trapping
MoS_2_ single crystal	1.0 ± 0.5 (19%)		28 ± 9 (27%)	1900 ± 265 (54%)
MoS_2_-pentacene heterojunction	**1.0** (18%)	21 ± 3 (34%)		**1900** (48%)

a Bold values are fixed during the fit for the heterojunction.

For the MoS_2_-pentacene heterojunction results, we follow a similar fitting method applied by Homan *et al*.,^15^ in which decay components that are not affected by the presence of pentacene are fixed, such as ultrafast defect-assisted decay and the slow decay components. The new intermediate component, with a decay time of 21 ± 3 ps, then is associated with the hole transfer from pentacene to the MoS_2_ crystal (see ref (15) for schematic diagrams illustrating the corresponding decay processes). This new component accounts for 34% of the fit, to be compared to the previous intermediate fraction of 26%. We were not able to detect any new component associated with slow recombination processes of transferred holes with excess electrons in MoS_2_ on the scale of a few nanoseconds, which most probably fall outside our time window of 2 ns used in the experiments. The exciton decay characteristics of MoS_2_ crystals covered by the pentacene thin film are found to be quite similar to those of uncovered MoS_2_. Notably, the time scale for exciton dissociation at the MoS_2_-pentacene interface of 21 ± 3 ps is slow compared to the previously reported hole transfer time of 6.7 ps for pentacene grown on MoS_2_ on SiO_2_/Si.^15^ The decay process of the B-exciton, which is presented in Figure 4b, is similarly not significantly affected by the presence of the pentacene film: All the fitted decay components (see Supporting Information) are very similar and agree within the error bar for both the MoS_2_ and MoS_2_-pentacene samples in our measurements. Figure 4b shows the data without fitted time traces, and the overlapping data sets clearly show the same type of behavior for both systems.

There are several possible explanations for the different exciton dynamics in our MoS_2_-pentacene samples fabricated by layer transfer as compared to the observations of Homan *et al*.^15^ The pentacene layers grown by physical vapor deposition on water-soluble poly(acrylic acid) are highly crystalline, as has been confirmed by X-ray diffraction measurements performed on similar samples both before and after layer transfer.^6^ Consequently, the MoS_2_-pentacene interface features pentacene molecules that are “standing up” as they would on the growth substrate. In contrast, disordered MoS_2_-pentacene interfaces formed by physical vapor deposition of pentacene on MoS_2_^15^ are expected to exhibit molecules that are more randomly oriented, including molecules that are lying flat on the substrate. Several experimental studies indeed have shown that molecular monolayers of pentacene deposited onto MoS_2_ comprise molecules that adopt such an orientation on the surface.^38,39^ Since exciton dissociation by hole transfer relies on the electronic coupling between the π- and π*-orbitals of pentacene and the MoS_2_ bands, the increased overlap of the electronic wave functions for molecules that are lying flat on MoS_2_ would indeed give rise to faster exciton decay.

In addition, the substrates on which the MoS_2_-pentacene layers reside may play a role. Our MoS_2_-pentacene heterojunctions were transferred onto gold substrates, while in the work of Homan *et al.* quartz substrates were used. For MoS_2_ gold interfaces, Fermi level pinning has been observed by us previously.^40^ According to a recent publication,^41^ the bandgap of MoS_2_ and WSe_2_ is also somewhat affected by the substrate: The band gap of MoS_2_ is decreased by 0.21 eV and that of WSe_2_ by 0.16 eV when the monolayers are located on Au substrates. These effects might lead to a different alignment of MoS_2_ and pentacene energy levels as compared to MoS_2_-pentacene heterojunctions on insulating quartz substrates. Huang *et al.* reported that the presence of a gold substrate modifies the charge distribution in MoS_2_.^42^ All of the above could affect the hole transfer rates at the MoS_2_/pentacene interface. We would like to stress, however, that in our experiments we used UV/ozone-treated Au substrates exhibiting a thin surface oxide, which decreases the aforementioned effects of gold on the TMDC monolayers significantly. For a strongly coupled Au/MoS_2_ system, photoexcited states in MoS_2_ could decay via interactions with the gold substrate,^43^ which then could affect the exciton decay behavior in samples both with and without pentacene. Since the exciton decay dynamics observed for our Au/MoS_2_ samples are quite similar to the findings of Homan *et al.*, who studied MoS_2_ on SiO_2_, this mechanism most probably does not play a major role in our experiments, and it can be concluded that the coupling between Au and MoS_2_ is rather weak.

### 2.3 Tungsten Diselenide (WSe_2_)

**Figure 5:**
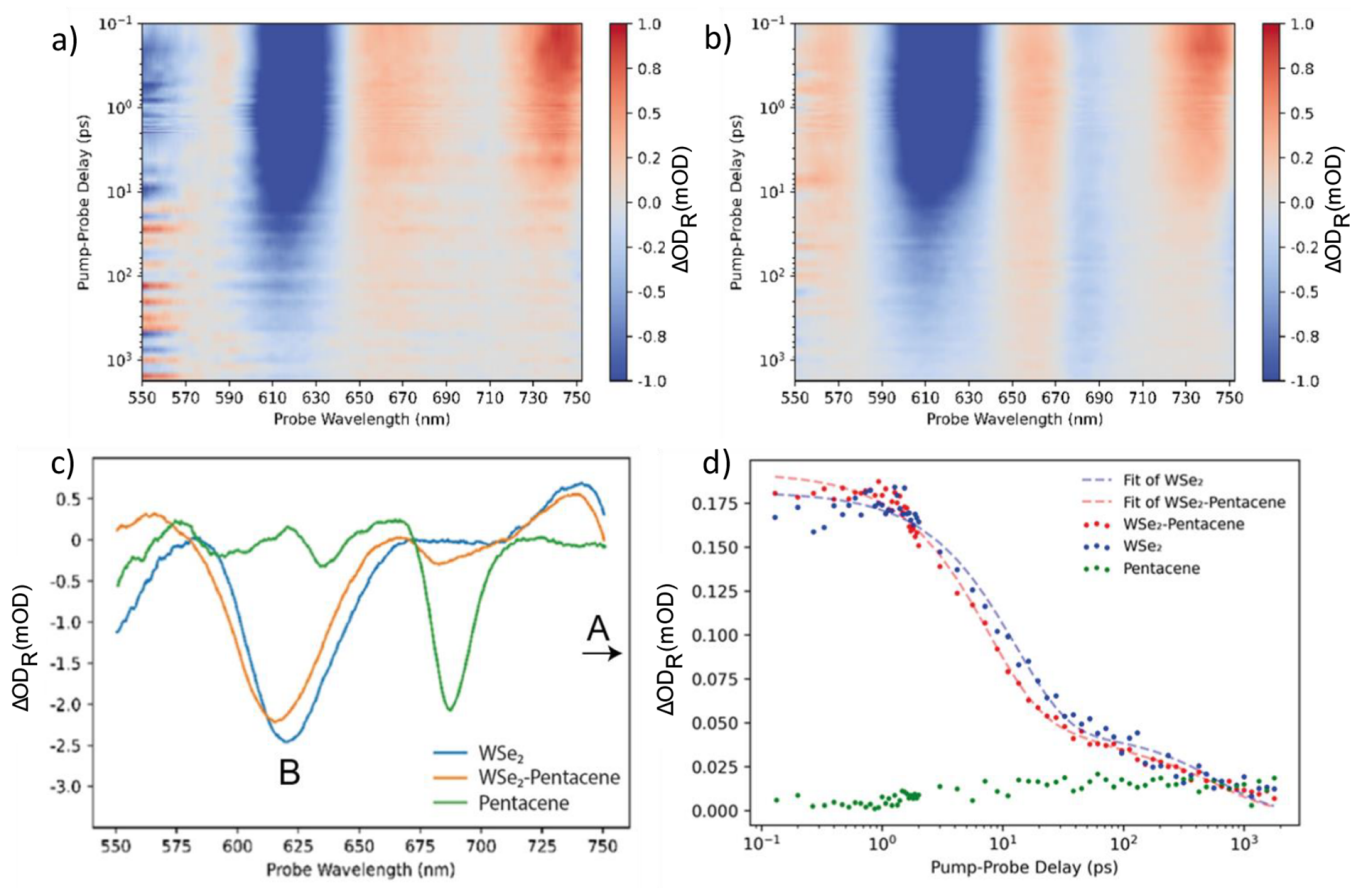
Transient reflection spectra of (a) WSe_2_ monolayer crystals transferred to a gold substrate and (b) WSe_2_ monolayer crystals covered by a pentacene thin film on the same substrate. A pentacene bleach peak appears at 688 nm in (b). (c) Transient reflection spectra of WSe_2_ monolayer, WSe_2_-pentacene junction and pentacene thin film, all on gold substrates, probed at 0.53 ps. (d) Normalized decay traces of B-excitons in a WSe_2_ monolayer compared to a WSe_2_-pentacene junction probed at 618 nm, both on gold substrates.

The second part of our work is dedicated to tungsten diselenide (WSe_2_) monolayer crystals compared to WSe_2_-pentacene heterojunctions. The pentacene layer is nominally the same as in the case of MoS_2_: a 20 nm thick continuous film. The WSe_2_ and pentacene layers again have been transferred onto a gold substrate. Our TRS measurements of WSe_2_ single crystals, shown in Figure 5a, show a bleach peak at 610 nm for the B-exciton and a positive signal near the expected wavelength of the A-exciton above 730 nm. The peak position of the B-exciton is in agreement with previous publications.^18,19^ The positive signal might be related to a photoinduced absorption feature of an excited state. In addition, excitonic features could sometimes produce positive signals instead of bleaching due to broadening of the absorption spectra after excitation by the pump pulse.^44^ The positive signal appears at the border of the studied spectral window, and hence its temporal evolution cannot be traced accurately. Figure 5b shows TRS data for the WSe_2_-pentacene junction. Besides the just discussed features of WSe_2_, one can easily recognize a bleach peak of pentacene at 688 nm.

Figure 5c shows the TRS spectra of a WSe_2_ monolayer (orange) and a WSe_2_-pentacene heterojunction (green), as well as a pentacene thin film (all on Au substrates). The spectra are recorded at 0.53 ps after excitation of the system by the pump pulse. The B-exciton peak is experiencing a slight blue shift in the presence of pentacene, to 615 nm from 621 nm for the WSe_2_-only sample. The observed shift might be related to a reduction of the exciton binding energy due to screening effects, as has been predicted for WSe_2_ encapsulated by hexagonal boron nitride.^45^

The most interesting region of the measured TRS data is associated with the B-excitons of WSe_2_ as was mentioned earlier. In this section, we repeat the same procedure of fitting an exponential decay function described above to WSe_2_ B-excitons for the two different samples. The exciton behavior in monolayer crystals of WSe_2_ is not as well studied as is the case for MoS_2_. A number of previous works focused attention on A- and C-excitons in few layer and bulk WSe_2_, mostly studying the ultrafast component <1 ps.^17−20^ He *et al*.^46^ provide some information on lifetimes of excitons in WSe_2_ monolayers. For the uncovered WSe_2_ monolayer, the decay traces of the B-excitons in our TRS measurements are well described by just two exponential components (see Figure 5d). Using a two exponential fit is not a unique way of studying the decay dynamics in TMDC monolayers and has been already implemented previously in work on MoSe_2_ monolayers.^47^ We do not detect a sub-picosecond decay component corresponding to ultrafast carrier trapping associated with defects in the single crystal, as found for MoS_2_. Previously, we reported that similarly prepared monolayers of WSe_2_ on metallic substrates feature vacuum level alignment according to the Schottky–Mott limit, unlike MoS_2_ layers for which Fermi level pinning is observed.^40^ This different energy level alignment points to a considerably lower defect density in WSe_2_ as compared to MoS_2_, consistent with the absence of ultrafast defect-assisted trapping. The obtained values for *t*_2_ and *t*_3_ are 12 ± 1 ps and 624 ± 112 ps. The latter component corresponds to one-quarter of the weight in the biexponential fit. The decay traces of the WSe_2_-pentacene heterojunction require an extra component with *t*_1_ equal to 7 ± 1 ps to obtain a good fit, which we attribute to hole transfer from pentacene to WSe_2_. In the WSe_2_-pentacene case, *t*_2_ (12 ps) is kept fixed; keeping both *t*_2_ and *t*_3_ or only *t*_3_ fixed did not provide any reasonable fitting results. It should be noted, however, that similar values for *t*_3_ as well as the weights of the slow decay component are found in the case of the heterojunction and uncovered WSe_2_ case.

**Table 2:** Fit Parameters for Decay Traces of B-Excitons in WSe_2_/WSe_2_-Pentacene Samples^a^

	*t*_1_ [ps] (A1)	*t*_2_ [ps] (A2)	*t*_3_ [ps] (A3)
	h^+^ transfer	exciton–phonon scattering	radiative recombination and e^–^ trapping
WSe_2_ single crystal		12 ± 1 (75%)	624 ± 112 (25%)
WSe_2_-pentacene heterojunction	7 ± 1 (39%)	**12 (40%)**	705 ± 95 (20%)

a Bold values are fixed during the fit for the heterojunction.

In addition, the decay of A-excitons in WSe_2_ was measured with a silicon detector using spectral filters only, by means of lock-in detection resulting in better S/N in the data while losing some spectral resolution. The filter used to obtain the data is a FB700-40, i.e., with a 40 nm range centered at 700 nm (Supporting Information 1). Using this filter is suitable for detecting the temporal evolution of the A-exciton signal because even though it covers a rather large portion of the spectrum, there is no (ultra)fast decay of other excited states, e.g., of pentacene, which could negatively impact the data, such that the information that we obtain derives from the A-excitons in WSe_2_. The behavior of the A-exciton is noticeably different from that of the B-exciton presented earlier. The decay trace of the uncovered WSe_2_ layer was fitted with a three exponential decay function comprising fast (8 ± 1 ps), intermediate (32 ± 6 ps), and slow (401 ± 35 ps) components. Again, an ultrafast sub-picosecond component is absent as in the case of the WSe_2_ B-exciton. The graph representing the fitting result can be found in the Supporting Information (Figure S7).

It turned out that it was not possible to follow a similar method for fitting for the decay trace of the WSe_2_-pentacene heterojunction as in the previous cases because fixing any of the components resulted in a poor fit of the data. The fast component *t*_1_ in the WSe_2_-pentacene case decreased by about 20% to 6.6 ± 1.3 ps. A new component with a *t*_2_ of 17.3 ± 4 ps is attributed to the TMDC-pentacene interactions. Interestingly, the slow component decreased by more than a factor of 3 and became 125.1 ± 11.3, while the weight in the fit is almost not affected and stays at around one-fifth in both cases. Because of the new manner of fitting without any fixed components, we do not assign the time components to known processes as was done previously.

The different behavior of the A- versus B-excitons might indicate a different formation and decay process of the B-exciton compared to the A-exciton, possibly related to the different excitation energies and the availability of different decay channels. Previous work on WS_2_ and WSe_2_ also showed different decay dynamics for these exciton species.^48^ Another work showed a significant difference in differential transmission of A- and B-excitons in MoS_2_, which is explained by spectral Γ broadening. This also explains the amplitude difference for these two excitons.^28^ Noticeable differences in photoluminescence emission (intensity and profiles) of A- and B-excitons were reported and connected to the sample’s defect density.^49^

**Table 3:** Fit Parameters for Decay Traces of A-Excitons in WSe_2_/WSe_2_-Pentacene Samples

	*t*_1_ [ps] (A1)	*t*_2_ [ps] (A2)	*t*_3_ [ps] (A3)	*t*_4_ [ps] (A4)
WSe_2_ single crystal	8.1 ± 0.9 (56%)		32.2 ± 5.6 (24%)	401.4 ± 34.8 (20%)
WSe_2_-pentacene heterojunction	6.6 ± 1.3 (54%)	17.3 ± 4.9 (29%)		125.1 ± 11.3 (17%)

## 3 Conclusion

We studied exciton dynamics in van der Waals heterojunctions of MoS_2_-pentacene and WSe_2_-pentacene. We have found that in the case of MoS_2_-pentacene heterojunctions the presence of the pentacene film has a moderate influence on the decay kinetics of A- and B-excitons. The A exciton was mostly affected by the presence of the pentacene film, exhibiting a 21 ± 3 ps decay component associated with hole transfer from MoS_2_ to pentacene. Our results are noticeably different compared to a previous report on a similar structure, i.e., pentacene grown on MoS_2_ on SiO_2_,^15^ showing a faster hole transfer on the time scale of 6.7 ps. Several factors contribute to this, in particular, the different molecular orientations in transferred versus vapor deposited pentacene films, and the utilization of a different substrate (gold in this work versus SiO_2_ substrate). The exciton dynamics could be affected by a different energy level alignment at the MoS_2_-pentacene interface, due to the Fermi level of MoS_2_ being pinned to that of the gold substrate.^40^ In the case of WSe_2_-pentacene heterojunctions, the A- and B-excitons showed different decay schemes. The B-exciton signal of the WSe_2_ monolayer was fitted with a two-exponential decay function, with 12 and 624 ps components. In the presence of pentacene, the best fit was obtained by adding a fast 7 ps component associated with hole transfer, while keeping the 12 ps component fixed and changing the slow component to 705 ps. The A exciton decay was best described by fitting of a three-exponential decay function. The presence of pentacene again resulted in overall faster decay of the WSe_2_ A-exciton. It was however not possible to assign individual decay components unambiguously to known processes because fixing the decay components did not provide reasonable fits. We believe that our results on TMDC-OS heterojunctions will help to stimulate future research and technical advancements in the area.

## 4 Methods and Materials

MoS_2_ and WSe_2_ monolayer single crystals were grown on thermally oxidized silicon substrates (Siltronix, oxide thickness 300 nm, roughness <0.2 nm RMS) by a modified CVD growth method in which a Knudsen-type effusion cell is used for the delivery of sulfur and selenium precursors.^21,23^

For heterostructure fabrication, the as-grown TMDs were transferred onto a suitable gold substrate. We have employed a poly(methyl methacrylate) (PMMA) assisted transfer protocol for the transfer.^21^ A PMMA layer of 200 nm (950 kDa, Allresist GmbH, AR-P 679.04) was spin coated onto the SiO_2_/Si substrate with CVD grown TMD crystals. Then the substrate was kept floating on top of a bath of KOH solution (85%, Carl Roth) to etch away the SiO_2_ layer and to release the TMD crystals supported by PMMA followed by washing several times with ultrapure water (18.2 MΩcm, Membrapure) to remove any residual KOH. Then the PMMA supported monolayer TMD was placed on the target substrate and baked at 90 °C for 10 min, followed by immersion in acetone for 2 h to remove the PMMA support, followed by rinsing with isopropyl alcohol.

A 50 nm thick poly(acrylic acid) (PAA) layer was spin coated on a borosilicate glass substrate as water-soluble sacrificial layer. Then, 20 nm thick pentacene was evaporated on PAA with a deposition rate of 0.1 Å s^–1^, while the substrate was held at room temperature. After the pentacene deposition, the highly ordered organic nanosheet was delaminated by dissolving the PAA layer by side injection of water from a reservoir.^6^ The floating nanosheet was transferred to a DI beaker to ease the transfer onto the target substrate. In parallel, the target substrate with transferred MoS_2_ was immersed into acetone for 2 h to remove the PMMA supporting layer prior to nanosheet transfer. Then, the floating nanosheet was picked up by the target substrate and carefully dried under ambient conditions.

The process of transient absorption/reflectivity spectroscopy was conducted by a dedicated system at the research institute ELI-ALPS (Szeged, Hungary). The setup consists of a high speed (2000 spectra/s) fiber optic spectrometer and a laser driving the experiments. The laser system is a few-optical cycle ytterbium fiber chirped-pulse amplifier system that generates 30 fs pulses, with a 100 μJ pulse energy, at a repetition rate of 100 kHz centered at 1030 nm. The schematic layout, a more detailed description, and validation experiment of the custom-built transient absorption/reflection spectrometer are described in Supporting Information 1. The generated laser pulse is split into a 80/20 ratio where 80% of the beam is used for the pump beam generation, and the remaining 20% generates the probe beam. The pump beam is frequency doubled on a Type-I BBO crystal (Eksma optics, 2 mm) resulting in a 515 nm center wavelength pulse. The pump-beam was focused on the sample surface with an excitation fluence of 0.7 mJ cm^–2^. The probe beam was focused on the sample surface into a 60 μm × 50 μm spot with a 10 μJ cm^–2^ fluence ensuring that the probe beam focus was smaller than the pump beam focus for optimal signal levels.

The obtained data were processed by a specially tuned Python script for background subtraction (Supporting Information 2). Spectral regions between the bleach peaks were adopted as reference points according to which background was subtracted appropriately. An example of the raw versus processed data, as well as the code, can be found in Supporting Information 2. The transient reflection spectra in images 3 and 6 were not additionally processed. Decay kinetic traces were obtained by direct averaging over a specified wavelength window without additional processing. The process of fitting the three-exponential decay function to study the exciton decay constants was compiled in a Python script utilizing the sklearn library including a least-squares error method.

## Supporting Information Available

Experimental details of transient absorption/reflection spectroscopy, transient reflection spectra of mechanically exfoliated MoS_2_ for validation of the setup, a description of Python scripts for data processing, and fits of decay traces of B-excitons in MoS_2_ and MoS_2_-pentacene samples (PDF)
