# Supplementary material for: Exciton Dynamics in MoS2-Pentacene and WSe2-Pentacene Heterojunctions
Source: ACS Nano. 2022 Sep 30;16(10):16668–76. doi: 10.1021/acsnano.2c06144 (PMC9620401; doi:10.1021/acsnano.2c06144)
Supplement: Supplementary file 1 — nn2c06144_si_001.pdf [file nn2c06144_si_001.pdf]

Exciton dynamics in MoS_2-pentacene and WSe_2-pentacene heterojunctions

Supporting Information

Pavel A. Markeev[1,*], Emad Najafidehaghani[2], Gergely F. Samu[3], Krisztina Sarosi[3], Sirri Batuhan Kalkan[4], Ziyang Gan[2], Antony George[2], Veronika Reisner[4], Karoly Mogyorosi[3], Viktor Chikan[3,5], Bert Nickel[4], Andrey Turchanin[2] and Michel P. de Jong[1]

1. MESA+ Institute for Nanotechnology, University of Twente, 7500 AE Enschede, The Netherlands.
2. Institute of Physical Chemistry, Abbe Center of Photonics, Friedrich Schiller University, 07743 Jena, Germany.
3. ELI-ALPS, ELI-HU Non-Profit Ltd., Wolfgang Sandner 3, Szeged H-6728, Hungary.
4. Faculty of Physics and CeNS, Ludwig-Maximilians-Universität, Geschwister-Scholl-Platz 1, 80539 Munich, Germany.
5. Dept. of Chemistry, Kansas State University, 213 CBC Building, Manhattan, KS 66506-0401, USA

* Corresponding author: p.markeev@utwente.nl

1. Experimental details of transient absorption/reflection spectroscopy

The transient absorption/reflectivity setup consists of a spectrometer and a laser driving its experiments. The driving laser system is a few-optical cycle ytterbium fiber chirped-pulse amplifier system that generates 30 fs pulses, with a 100 μJ pulse energy, at a repetition rate of 100 kHz centered at 1030 nm (see Figure S1 for spectrum and autocorrelation). This laser system is located at the ELI-ALPS laser facility [1]. The schematic layout, a more detailed description, and a validation experiment of the custom-built transient absorption/reflection spectrometer are shown in Figures S2-3.

As shown in Figure S2, the incoming laser is split in a 80/20 ratio and 80% of the beam is used for the pump beam generation while the remaining 20% generates the probe beam. The incoming laser contained negative chirp to obtain optimal temporal length of the pump. The pump beam is frequency doubled on a Type-I BBO crystal (Eksma optics, 2mm) resulting in a 515 nm center wavelength pulse. The remaining 20% of the fundamental was used to generate a white light continuum via focusing the beam on a c-cut [111] sapphire crystal. The residual fundamental from the white light was removed with the help of an IR blocking filter. In these experiments, the pump-beam was focused on the sample surface with an excitation fluence of 0.7 mJ cm^{-2}. In all cases before measurements, the power density of the pump was monitored to ensure that similar amounts of charge carriers were generated in each sample. In addition, the transient reflectivity of a known bulk sample was used to verify that the exciton dynamics under these conditions were similar to the observations in the literature [2]. The probe beam was focused on the sample surface into a 60×50 μm^2 spot with a 10 μJ cm^{-2} fluence ensuring that the probe beam focus was smaller than the pump beam focus for optimal signal levels. The samples deposited on the various substrates were placed on a *x-y* controlled sample holder ensuring sufficient

spatial overlap of the pump and probe beams. During the measurements the samples were continuously purged with a flow of N_2, to avoid sample degradation.

During the experiment, two distinct measurements were performed to monitor the transient reflection of the samples. For spectra acquisition, a fast fiber optic spectrometer (Ocean Insight FX) was used to monitor the spectral changes in the 200 -1100 nm range. A mechanical chopper operated at 2000 Hz was used to record pump-on and -off spectra, and was synchronized to the spectrophotometer triggered at 4000 Hz (with a 30 µs integration time averaging 3 pulses at a time from the 100 kHz laser). 10000 spectra were recorded and used to calculate the ΔOD_R traces. To record the kinetic traces in a fixed spectral range, a photodiode (Thorlabs PDA100A-EC) coupled with a lock-in amplifier (Stanford Research Systems SR865A) was used yielding better signal to noise ratio in the data than the spectral measurements. The white light was filtered with appropriate bandpass filters to match the transient response of each sample. The spectra acquisition, data analysis and visualization were done by custom MATLAB and LabVIEW programs. For a typical measurement, several data points were collected and averaged to ensure good representation for the sample.

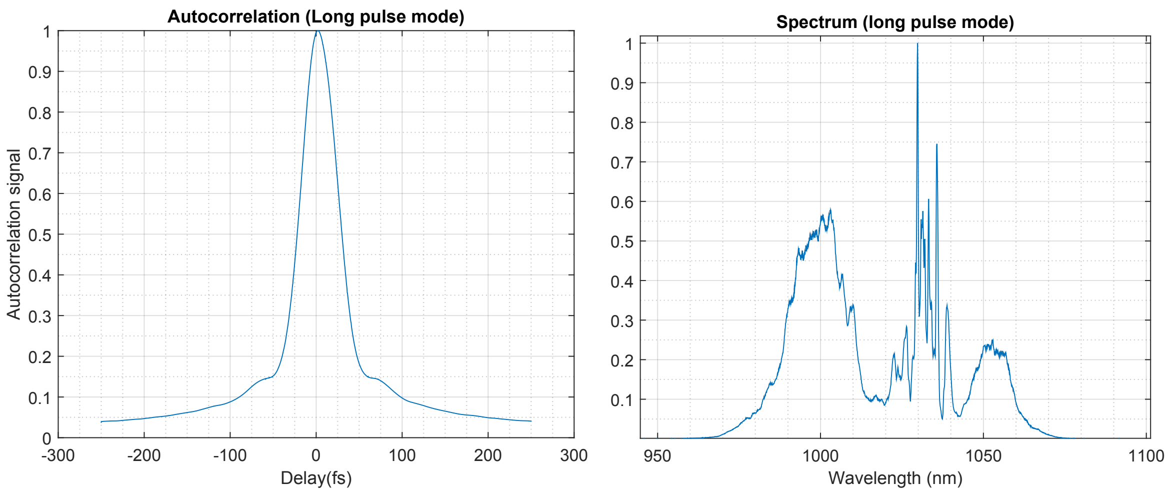

Figure S1. *Autocorrelation and output spectrum of HR-1 laser.*

When the laser beam from the HR-1 laser enters (from the right in Fig. S2) the TAS/TRS enclosure immediately an iris (RI1) blocks out the outer parts of the beam, so that only the core of the laser beam is used for the experiments. As mentioned previously the incoming laser beam is split in an 80/20 ratio with a beam splitter (BS). 80% of the beam is used for the generation of the pump pulses. It is focused down (L) on a Type-I BBO crystal (Eksma optics, 2mm) resulting in a 515 nm center wavelength pulse. A mechanical chopper is placed in a sufficiently narrow part of the beam, before the second harmonic generation. After collimation (L) of the pump pulse an iris (RI3) is used to further block outer parts of the beam. Through several silver mirrors (SM) the pump pulse is passed through a delay stage (DS) mounted with a retroreflector (RR) to induce the time-delay between the pump-probe pulses. A final focusing (L) of the pump-beam is carrier out right in front of the sample. The remaining 20% of the fundamental was used to generate a white light continuum via focusing the beam on a c-cut [111] sapphire crystal (SAP). The quality and stability of the white light continuum was fine-tuned through the opening/closing of the iris (R1) in the beam path and through the rotation/repositioning of the sapphire crystal. The generated white light was collected with a parabolic mirror (PM) and passed through an IR blocking filter, to remove any residuals from the fundamental. The probe-beam was spatially overlapped with the pump beam on the sample surface with a half-silver mirror (HSM).

The reflection from the sample surface was collected with a silver mirror from which the pump-pulse was separated using a pinhole (50 µm) placed in the beam-path. Using flip-mirrors switching between the detection modes (Lock-in detection or spectrophotometric detection) was achieved. It is also worth mentioning that the developed setup is capable to measure in transmission mode as well.

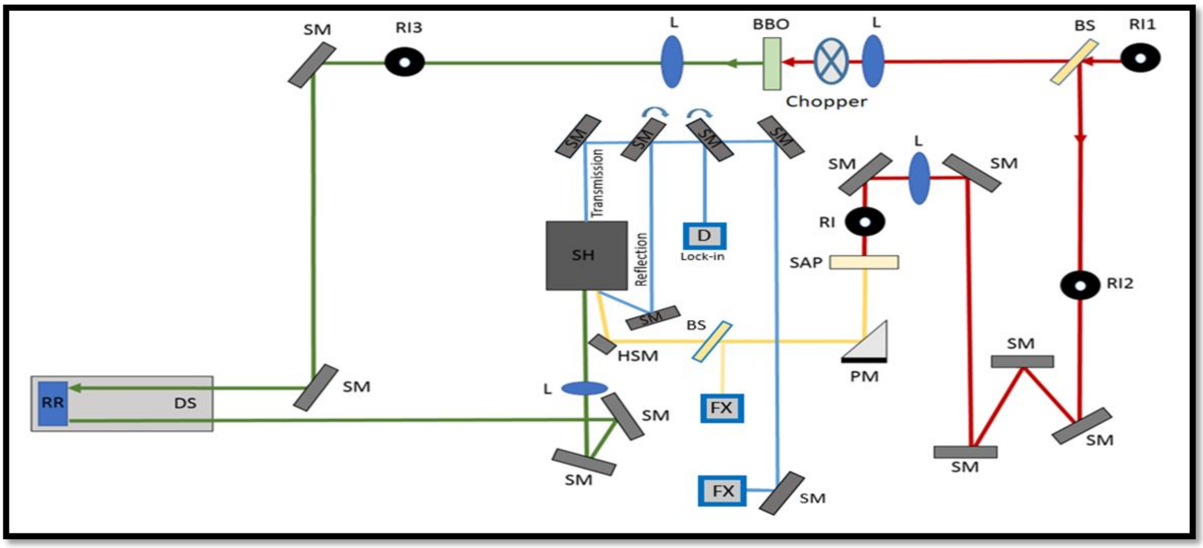

Figure S2. *The schematic layout of the custom-built transient absorption/reflection setup.*

Validation of the setup

Mechanically exfoliated MoS_2 samples on glass substrates were used to validate the transient absorption/reflection setup. We recorded the decay trace of the B-exciton bleach signal at 612 nm under an excitation fluence of 0.7 mJ cm^{-2} with a pump pulse centered at 515 nm. The decay trace was fitted with a triexponential function [2], [3], and the fitting parameters are summarized in Table S1. The fastest component is assigned to quenching of the MoS_2 exciton by carrier trapping [4], the second component to exciton−phonon scattering processes [3], and the longest component is consistent with previously reported time constants for radiative recombination of the exciton (250−850 ps) [2][3]. The relative weight of these processes, however, are different compared to previous reports, which might be the cause of the different quality of the samples or the used pump intensity in our study.

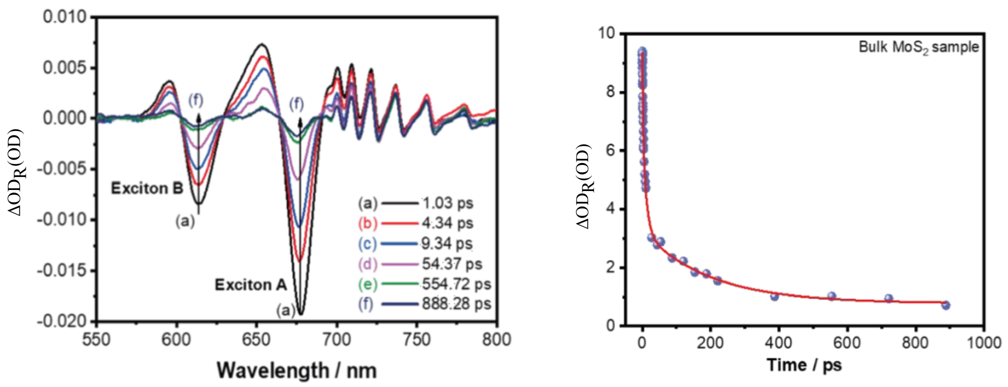

Figure S3. *Transient reflection spectra recorded for mechanically exfoliated bulk* MoS_2 *sample used to validate the setup. Decay curve recorded at 615 nm for the same mechanically exfoliated bulk-*MoS_2 *flake with an excitation fluence of 0.7 mJ* cm^{-2}*. The decay trace was fitted with a tri-exponential decay as described in reference* [2]. *The fitting parameters are summarized in* ***Table S1***.

Table S1. Summary of fitting parameters from the triexponential fitting of the decay trace shown in Figure S3 for a bulk MoS_2 sample. The parameters are compared with literature values from reference [2].

Fitted parameter	Bulk MoS_2 sample	Reference S1
A_1	0.0024 (28%)	0.47 (47%)
t_1 / ps (carrier trapping)	1.97	0.67
A_2	0.0038 (44%)	0.35 (35%)
t_2 / ps (exciton-phonon scattering)	10.9	15.8
A_3	0.0025 (29%)	0.18 (18%)
t_3 / ps (radiative recombination)	198	431

2. Data processing python scripts

2.1 Background subtraction

The following script was implemented to reduce intensity variations as can be seen on the image S4 before and after processing. Data processing for exciton dynamics without subtraction of intensity variations was not possible. The code, which can be found at https://github.com/pmarkeev/TRS_TMDC_excitons.git, is written in a Spyder environment and runs on Python 3.8.

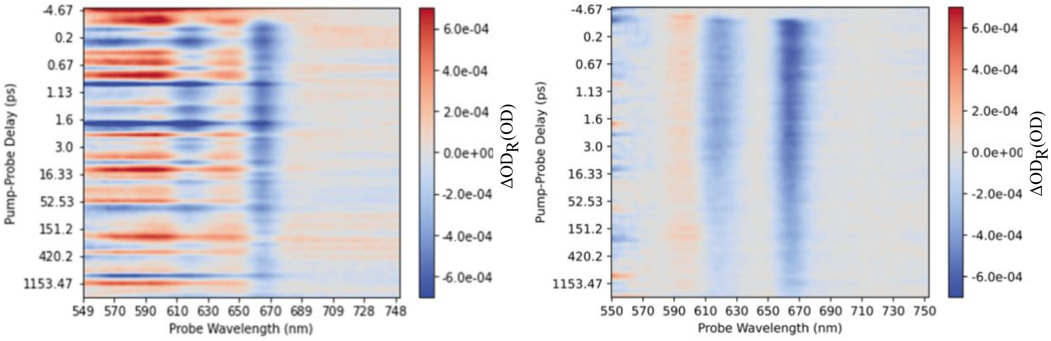

Figure S4. *Representative example of original transient reflection data before (left) and after (right) processing.*

2.2 Exponential decay fitting

A second script was used to fit a 3-exponential decay function to the measured decay traces of excitons. The code allows to obtain 3 time constants and their fractional amplitudes. It also can be found at https://github.com/pmarkeev/TRS_TMDC_excitons.git

3. MoS_2 B exciton fitting results

The results of fitting a 3-exponential decay function to the MoS_2-only monolayer B-exciton can be found in the Table S2. An ultrafast component is absent, so only fast, intermediate and slow components were fitted. The fitting with 3 exponential decay functions has a much larger error in comparison to the results for A-exciton. For the heterojunction case, the component associated with the presence of pentacene (t_2) is insignificantly decreased. It was concluded that there is no significant effect of the pentacene because both values for t_2 and t_3 are within the error. In this case t_1 and t_4 were kept fixed from the MoS_2 monolayer fit.

Table S2. Fit parameters for decay traces of B-excitons in MoS_2 and MoS_2-pentacene samples. Bold values are fixed for the heterojunction.

	t_1 [ps] (A1)	t_2 [ps] (A2)	t_3 [ps] (A3)	t_4 [ps] (A4)
MoS_2 monocrystal	11±9 (28%)		71±51 (40%)	2000±683 (32%)
MoS_2-Pentacene heterojunction	**11** (38%)	61±23 (24%)		**2000** (38%)

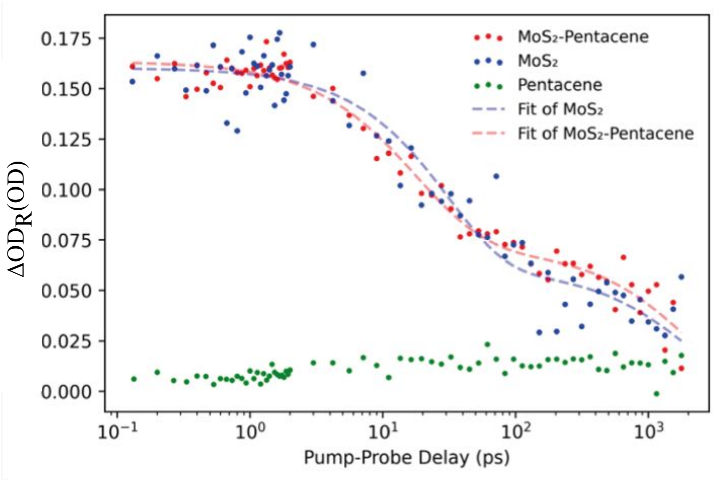

Figure S5. *Normalized decay traces of B-excitons in a MoS_2 monolayer compared to a MoS_2-pentacene junction probed at 615 nm.*

4. PEEM image of the studied samples.

Figure S6.a(b) shows a PEEM image of a MoS_2-(WSe_2-) pentacene sample, obtained at the Eli-Alps laser institute with a NanoEsca end station and He(I) radiation with an energy of 21.2 eV.

a) b)

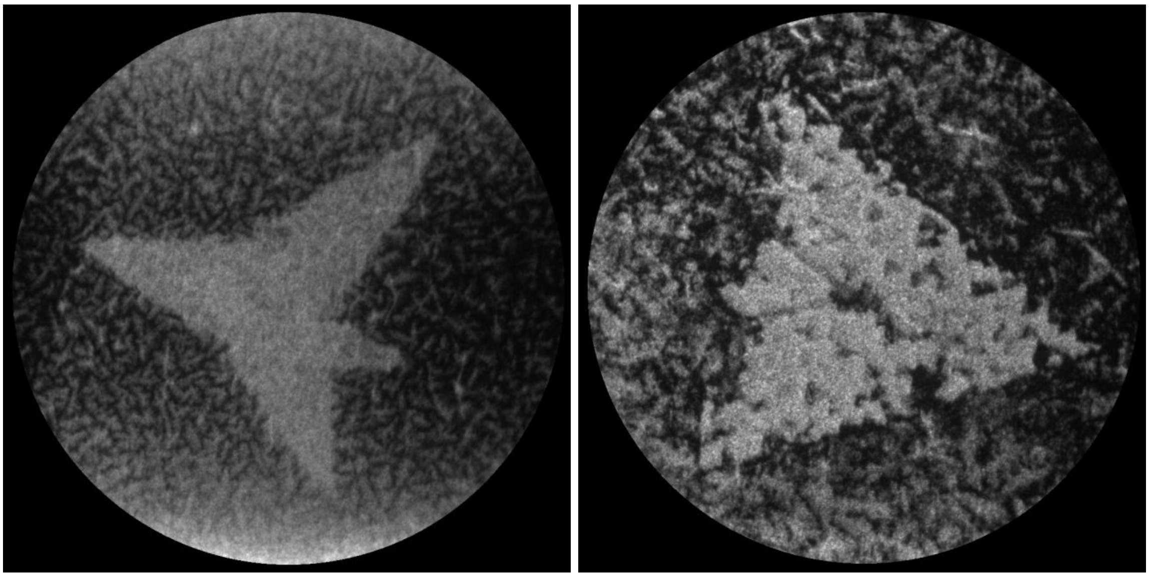

Figure S6. *PEEM images of a) MoS2-pentacene, field of view (FoV) = 74 µm; b) WSe2-pentacene FoV = 40 µm.*

5. Fitting results of the decay traces of A exciton in WSe_2 and WSe_2-pentacene samples.

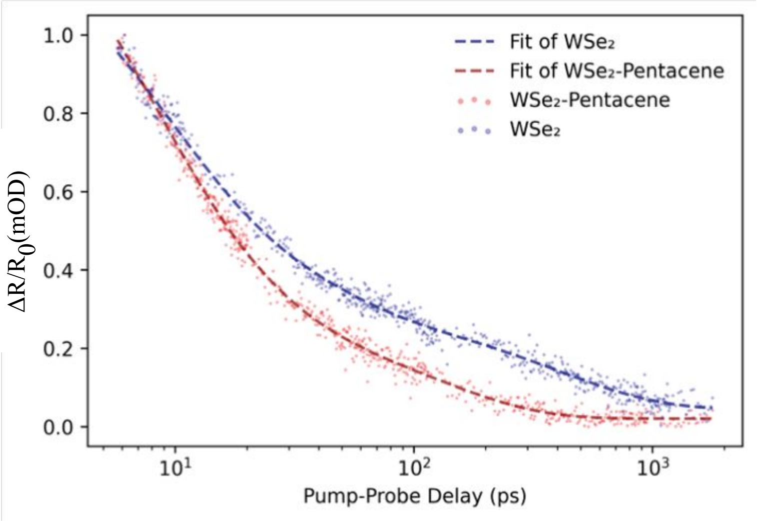

Figure S7. *Normalized decay traces of A excitons in a WSe_2 monolayer compared to a WSe_2-pentacene junction probed with a lock-in amplifier in 700±20 nm wavelength range. Note that the y-axis is $\Delta R/R_0 = ((I_{R,exc}-I_{R,noexc})/I_{R,noexc})$*

List of references

[1] Charalambidis, Dimitris, et al. "The extreme light infrastructure—Attosecond light pulse source (ELI-ALPS) project." *Progress in Ultrafast Intense Laser Science XIII*. Springer, Cham, **2017**. 181-218.

[2] S. B. Homan, V. K. Sangwan, I. Balla, H. Bergeron, E. A. Weiss, and M. C. Hersam, "Ultrafast exciton dissociation and long-lived charge separation in a photovoltaic pentacene-MoS_2 van der Waals heterojunction," *Nano Lett.*, **2017**, vol. 17, no. 1, pp. 164–169.

[3] H. Shi *et al.*, "Exciton dynamics in suspended monolayer and few-layer MoS2 2D crystals," *ACS Nano*, **2013**, vol. 7, no. 2, pp. 1072–1080.

[4] H. Wang, C. Zhang, and F. Rana, "Ultrafast Dynamics of Defect-Assisted Electron–Hole Recombination in Monolayer MoS 2," *Nano Lett.*, **2015**, vol. 15, no. 1, pp. 339–345.
